# CAR-T cells for pediatric malignancies: Past, present, future and nursing implications

**DOI:** 10.1016/j.apjon.2023.100281

**Published:** 2023-08-03

**Authors:** Colleen Callahan, Lauren Haas, Laura Smith

**Affiliations:** Division of Oncology, The Children's Hospital of Philadelphia, Philadelphia, USA

**Keywords:** Pediatric chimeric antigen receptor-T cell therapy, Pediatric relapsed and refractory ALL, Nursing management

## Abstract

The treatment landscape for pediatric cancers over the last 11 years has undergone a dramatic change, especially with relapsed and refractory B-cell acute lymphoblastic leukemia (ALL), due to the introduction of chimeric antigen receptor-T (CAR-T) cell therapy. Because of the success of CAR-T cell therapy in patients with relapsed and refractory B-cell ALL, this promising therapy is undergoing trials in multiple other pediatric malignancies. This article will focus on the introduction of CAR-T cell therapy in pediatric B-cell ALL and discuss past and current trials. We will also discuss trials for CAR-T cell therapy in other pediatric malignancies. This information was gathered through a comprehensive literature review along with using first hand institutional experience. Due to the potential severe toxicities related to CAR-T cell therapy, safe practices and monitoring are key. These authors demonstrate that nurses have a profound responsibility in preparing and caring for patients and families, monitoring and managing side effects in these patients, ensuring that study guidelines are followed, and providing continuity for patients, families, and referring providers. Education of nurses is crucial for improved patient outcomes.

## Introduction

In April 2012, the treatment landscape for pediatric relapsed B-cell acute lymphoblastic leukemia (ALL) was transformed. Using targeted immunotherapy, the first pediatric patient with relapsed B-cell ALL was infused with anti-CD19 chimeric antigen receptor-T (CAR-T) cells.^1^ This patient remains in remission today. This unprecedented success has sparked increased research and trials using CAR-T cell products for relapsed and refractory B-cell ALL, along with other relapsed pediatric cancers such as T-cell ALL, acute myeloid leukemia (AML), lymphoma, and solid tumors, including neuroblastoma and brain tumors. As a result of the positive outcomes with CAR-T cell therapies for B-cell malignancies and other landmark immunotherapy studies, immunotherapy has become the 4th treatment modality for cancer, along with chemotherapy, radiation, and surgery.^2^

In patients with relapsed and refractory malignancies, cancer becomes more difficult to treat due to dose-limiting toxicities and chemotherapy resistance, indicating the need for novel therapies such as CAR-T cell therapy. Ongoing research in genetics, disease biology, and immunology has impacted the discovery of effective and less toxic targeted therapy in pediatric cancer.^3^

CAR-T cell therapy is a form of targeted immunotherapy that uses tumor-specific antigen recognition. Chimeric antigen receptors are customized receptors that target specific antigens on a malignant cell.^4^ A patient's own T cells are collected through apheresis and then undergo the CAR-T cell manufacturing process. During this phase, the T cells are genetically modified to express the CAR. Once manufacturing is complete, the CAR-T cells are ready to be infused into the patient. A goal of this therapy is for these engineered CAR-T cells to recognize and attack the target antigen, proliferate, and persist to provide long-term disease surveillance.^5^

CAR-T cells have shown remarkable clinical activity in B cell cancers, and the current Food and Drug Administration (FDA)-approved CAR-T cell product in pediatrics is directed at relapsed and refractory B cell ALL. With CAR-T cell therapy becoming more widely used and with the introduction of CAR-T cell trials in other pediatric cancers, nurses will be at the forefront of caring for these patients both inpatient and outpatient. They will be responsible for educating patients and their families and providing care to these patients during the CAR-T cell experience.

## Anti-CD19 CAR-T cell history

Pediatric ALL is the most common cause of cancer in children.^6^ Scientific and clinical research have contributed to cure rates greater than 80%.^7^ Unfortunately, some patients relapse and/or have refractory disease, and there is significant mortality in this population. There is an increased risk of morbidity with the addition of additional salvage therapies.^8^

Due to the risk of dose-limiting toxicities and chemotherapy resistance, research in targeted immunotherapy led to phase 1 and phase 2 CAR-T cell trials. A phase 1-2a single-center anti-CD19 CAR-T cell trial for pediatric and young adult patients with relapsed or refractory B-cell ALL showed promising results with a complete response (CR) rate of 93%.^9^ Based on these results, a phase 2 global, multisite trial for this population of patients was developed. This trial included 25 sites in 11 countries. Patients were enrolled and infused from 2015 to 2017. Median follow-up at 38.8 months showed that of 79 patients infused, there was an 82% overall remission rate, along with 3-year relapse-free survival (RFS) and overall survival rates of 52% and 63%, respectively. Favorable long-term safety was demonstrated.^8^

**Fig. 1:**
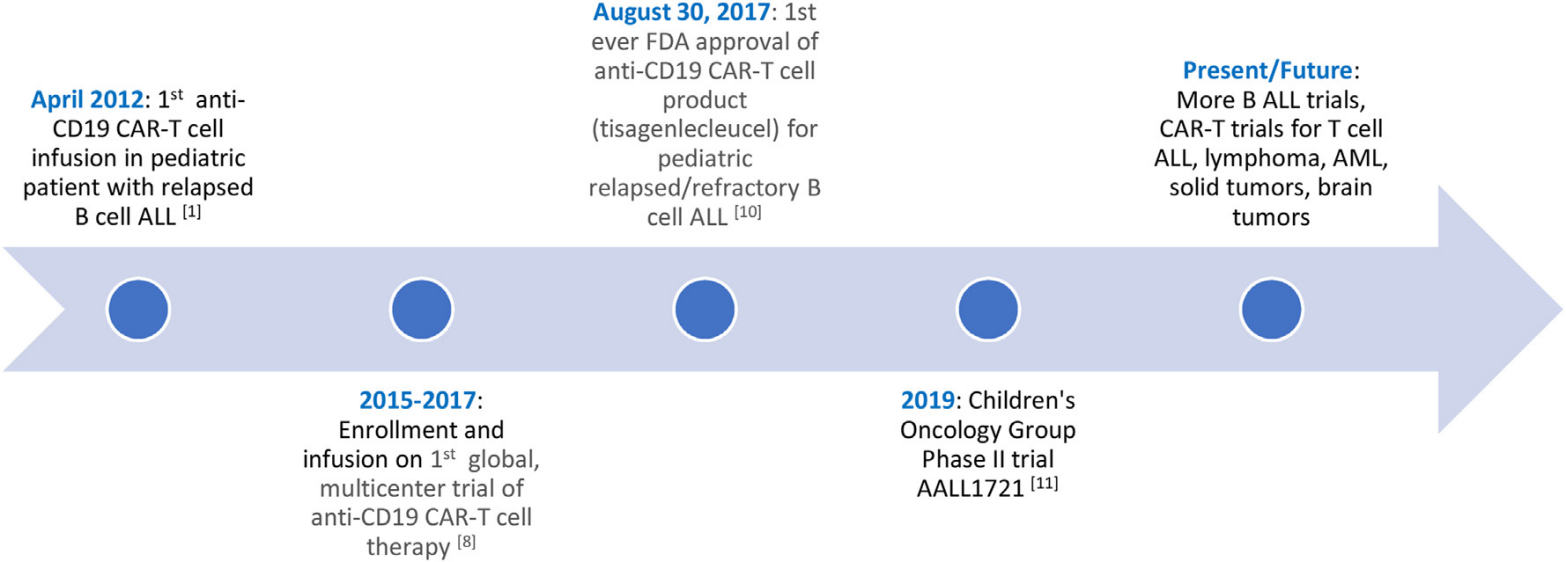
CAR-T cell timeline for pediatric malignancies. CAR-T, chimeric antigen receptor-T.

These pivotal phase 2 global, multisite trial response rates and safety results were integral in leading to FDA approval of tisagenlecleucel, the first commercialized anti-CD19 CAR-T cell product, in August 2017 for the treatment of pediatric relapsed or refractory B cell ALL. The first report of tisagenlecleucel in the real world shows similar response rates and safety compared with the phase 1-2 a single center trial and the phase 2 global, multisite trial. With a median follow-up of 13.4 months, the initial CR rate was 85.5% and the 12-month duration of response was 60.9%. Cytokine release syndrome (CRS), a systemic inflammatory response involving elevated cytokines in response to immune system activation, occurred in 55% of the patients. Immune effector cell-associated neurotoxicity syndrome (ICANS) was seen in 27% of patients.^10^ In the Children's Oncology Group, there is a phase 2, single-arm, multi-center trial looking at using tisagenlecleucel up front for high-risk pediatric patients who have positive minimal residual disease at the end of consolidation (Fig. 1).^11^

## B cell hematologic malignancies

### B cell ALL relapse post CAR-T cell therapy

Unfortunately, patients can relapse after anti-CD19-directed CAR-T cell therapy in one of two ways. A patient can relapse with CD19-positive B cell ALL, meaning that the ALL still expresses the CD19 antigen. This often happens when CAR-T cells have short-term persistence; therefore, disease surveillance is gone. Short term persistence can be related to immune-mediated rejection or CAR-T cell exhaustion. CD19-negative relapses can happen due to the loss of expression of CD19 on the tumor cell surface. Patient outcomes for B-cell ALL relapse post-anti-CD19 CAR-T cell therapy are poor.^12^ Investigating methods for B-cell ALL relapse following CAR-T cell therapy has led to newer CAR-T cell trials to address these issues.

### Humanized CAR-T cells

B cell aplasia, an on-target/off-tumour toxicity following CAR-T cell infusion, is used as a marker for CAR-T cell persistence. Loss of B-cell aplasia indicates loss of CAR-T cell persistence.^13^ Loss of CAR-T cell persistence is an issue for at least 25% of patients receiving CAR-T cell infusions.^12^ Patients with early loss of CAR-T cell persistence, indicated by B cell recovery during the first 6 months, have poor outcomes.^13^

Most CAR-T cells contain an extracellular antigen-binding domain that is derived from a murine monoclonal antibody, possibly leading to an antimurine immune response causing CAR-T cell rejection. Studies have been done and are ongoing looking at replacing the murine component with a humanized component to avoid this immune rejection and improve long-term persistence.^12^ A study done at one institution showed that humanized CAR-T cells can help patients achieve remission with long-term persistence. This CAR was based on the backbone of tisagenlecleucel. This study had 2 cohorts, retreatment, and CAR naïve. The retreatment cohort included patients who had received a prior murine CAR-T cell product and who had non-response, early B cell recovery, or CD19+ ​relapse. In the retreatment cohort, results showed an overall response rate (ORR) with patients having B cell aplasia at day 28 of 64%. RFS at month 12 was 74% and 58% at month 24. In the CAR naïve cohort, consisting of patients who never received a CAR-T cell product, the CR rate at day 28 was 98%, the RFS at month 12 was 84%, and 74% at month 24.^12^ These results indicate that this is a promising therapy.

### Universal CAR-T cells

For some patients, successful collection and manufacturing of T cells is extremely difficult due to multiple factors including heavy chemotherapy pretreatment, a high level of circulating blasts, and age at diagnosis. A study showed that naïve and early memory T cells in the collection product correlate with successful CAR-T cell performance and that deficits in naïve T cells can be seen in patients who have been heavily pretreated, contributing to harvesting and manufacturing failure. There is also a concern about the quality of T cells collected in patients with infant ALL.^14^ Other barriers to autologous CAR-T cell therapy include cost, length of time of manufacture, T cell dysfunction, and disease progression and death while awaiting manufacture.^15^ For patients with highly aggressive diseases, it is not always possible to successfully manufacture a product.

Universal CAR-T cells are products using T cells collected from healthy allogeneic donors. These cells are also referred to as “off-the-shelf” CAR-T cells. This is a promising therapy for patients who have had unsuccessful T cell collections or who have a highly aggressive disease and may have disease progression before an autologous CAR-T cell product is ready.^16^ Advantages of these cells include decreased cost, the ability to manufacture multiple doses from one donor, cells free of exposure to chemotherapy, and availability for immediate use.^17^ There are also a few disadvantages to off-the-shelf CAR-T cell products. Graft-versus-host disease (GVHD) is a risk as well as rejection of the universal CAR-T cells; therefore, these cells will not have the potential for persistence like autologous CAR-T cells. Intensification of the lymphodepleting chemotherapy by adding alemtuzumab, an anti-CD52 immunotherapy, is often used to condition the patient's immune system to minimize rejection of the CAR-T cells. Due to this immunosuppression, infection is a risk. Marrow suppression and prolonged cytopenias leading to viral reactivation are real possibilities.^17^

A universal CAR-T cell trial that enrolled patients aged 9 months to 62 years from 2016 to 2018 showed that universal CAR-T cell therapy is feasible. The median time from trial consent to beginning lymphodepletion was 11 days. This trial included 7 pediatric patients and 14 adult patients with relapsed or refractory B cell ALL. CRS was experienced by 91% of the patients, ICANS by 38%, and GVHD by 10%. Viral infections, including cytomegalovirus, adenovirus, human metapneumovirus, and BK virus, were seen in some patients. Sixty-seven percent achieved CR 28-days post CAR-T cell infusion, and 71% of the responding patients proceeded to stem cell transplant (SCT).^17^ Recently, results from another phase 1 universal CAR-T cell trial were published. This trial enrolled patients between the ages of 6 months and 18 years of age. Six patients were infused, and 4 were in remission and proceeded to SCT. In the immediate period post-CAR-T cell infusion, manageable CRS, ICANS, and viral reactivation were seen.^18^ More trials are needed to draw meaningful conclusions regarding efficacy, but both trials show that universal CAR-T cell therapy is safe, feasible, and has potential for patients for whom autologous CAR-T cell therapy is not a possibility.

### Anti-CD22 CAR-T cells

As previously mentioned, patients who relapse post anti-CD19 CAR-T cell therapy can relapse with CD19-negative disease. Evidence shows that around 50% of patients receiving anti-CD19 CAR-T cell therapies may relapse within the 1st year.^19^ Approximately 40% of these relapses are CD19 negative.^20^ CD19-negative relapses led to looking at alternative targets. These patients have limited treatment options and need alternative therapies. Since the advent of anti-CD19 immunotherapies, CD19-negative relapses have been seen as the main mechanism of resistance. Although the leukemia cells no longer express the CD19 antigen, CD22 expression is usually retained. CD22 is an antigen that is expressed in most cases of B-cell malignancies, and expression in normal tissue is limited to B cells.^21^ Anti-CD22 CAR-T cells are currently being trialed. The safety profile is similar to anti-CD19 CARs and anti-leukemia responses have been seen.

The results of a phase 1 anti-CD22 CAR-T cell trial were recently released. Fifty-eight patients were infused, 87.9% after receiving anti-CD19 CAR-T cells. CRS occurred in 86.2% of patients, and mild neurotoxicity occurred in 32.8% of patients. The complete remission rate was 70%. Seventy-five percent of these patients eventually experienced relapse. It was noted that patients who received prior CD22 targeted therapy had shorter remission durability and decreased MRD negative complete remission rates.^19^ This trial showed that these CAR-T cells can be an effective salvage regimen for patients who otherwise have limited treatment options.

### Dual CAR-T cells

Anti-CD22 CAR T cell trials for relapsed and refractory B cell ALL showing anti-leukemia responses have opened the door to using dual-targeting CAR-T cells. A central belief in the initial treatment of ALL is to use multi-agent chemotherapy to avoid relapse due to drug resistance.^21^ Using single antigen-targeting agents may lead to CD19 escape, which is a major challenge in CAR-T cell therapy. Dual CARs, targeting the CD19 and CD22 antigens, have been developed with the hope of overcoming CD19 antigen escape and improving outcomes.^22^ Dual CAR-T cells can be engineered in 4 different ways including: coadministration, bicistronic, cotransduction, and tandem.^23^ The optimal manufacturing and administration methods have not been determined and are still under investigation.

Dual CAR trials targeting both CD19 and CD22 have been done and show that there is no increased toxicity. Results from a dual CAR-T cell trial targeting CD19 and CD22 were recently released. Two hundred and twenty-five patients younger than 20 years were enrolled in a phase 2 trial, and 194 patients were infused. A complete response rate of 99% was seen. Event-free survival (EFS) at 12 months was 73.5%, and overall survival at 12 months was 87.7%. Improved EFS was seen with patients proceeding to SCT and in patients with B cell aplasia, indicating CAR-T cell persistence lasting longer than 6 months. CRS occurred in 88% of patients and ICANS in 20.9%. Three deaths occurred during CRS and/or ICANS.^22^ Results of this trial showed that this dual CAR-T cell product showed efficacy and helped patients achieve durable remissions.

### B cell non-hodgkin lymphoma (NHL)

Most pediatric patients with mature B-cell NHL have excellent prognoses with chemotherapy. Relapses are rare, but relapsed disease has decreased chemosensitivity and a dismal prognosis.^24^^,^^25^ CAR-T cell therapy is a promising novel therapy that can be used for this population of patients. Targets for CAR-T cell therapy for B cell NHL include CD19, CD20, and CD22 and there are current trials investigating the safety and efficacy.

Results from a phase 2 global, multicenter study using tisagenlecleucel in pediatric patients with relapsed or refractory B-cell NHL showed safety and efficacy. ORR was encouraging, although better in patients with large B-cell lymphoma (46%) vs. Burkitt lymphoma (20%). Neurologic events occurred in 27% of patients, and CRS occurred in 70% of patients.^26^

## Non-B cell hematologic malignancies

Developing successful CAR-T cell therapies for non-B-cell hematologic cancers has been more challenging. Some challenges have included finding tumor-specific antigens to target and avoiding severe, unacceptable on-target/off-tumor toxicities that attack normal, healthy cells. T cell aplasia as a result of anti-T cell ALL CAR-T cells and myeloid aplasia as a result of anti-AML CAR-T cells are examples of unacceptable long-term off-tumor toxicities.

### T cell ALL

Despite the success seen with CAR-T cell therapy for B-cell ALL, the development of successful strategies for T-cell ALL has been more difficult. T-cell ALL accounts for 10%–15% of cases of ALL.^27^ Relapsed and refractory T cell ALL is notoriously difficult to treat and has a dismal outcome with an overall survival rate of < 10%.^28^

There are unique challenges with CAR-T cells for T cell ALL including CAR-T cell product contamination with T lymphoblasts, CAR-T cell fratricide (the self-killing of CAR-T cells), and the on-target/off-tumor toxicity of T cell aplasia, leading to life threatening immunodeficiency.^20^^,^^29^

Early-stage trials are being done using autologous and universal CAR-T cells. A recent phase 1, human universal anti-CD7 CAR-T cell trial has shown some promising results. The use of a universal CAR-T cell from a healthy donor alleviates the problem of product contamination with leukemic cells. Twenty patients were infused. There were no dose-limiting toxicities. The CAR-T cells expanded, 90% of patients achieved a complete response (CR), and this was proven to be a safe therapy.^27^ Results support further investigation into CAR-T cells for relapsed and refractory T cell ALL. It is too early in this therapy to know if CAR-T cell therapy for T cell ALL is enough to be curative without going to SCT.^29^

### Acute myeloid leukemia

Relapsed and refractory AML remains challenging to treat due to chemotherapy resistance. The only potential curative therapy for this patient population is SCT; therefore, alternative therapies are needed for these patients.^20^ Challenges in CAR-T cell therapy for use in AML are similar to those in T ALL, specifically finding a targetable antigen that does not cause unacceptable toxicity. Most targetable antigens are found in normal myeloid progenitors. Destruction of these progenitors can lead to profound immunosuppression and life-threatening infections.^20^^,^^28^ Targets being studied for relapsed and refractory AML include CD33, CLL-1, and CD123. CD33 is expressed on the majority of AML cells, and the experience of using gemtuzumab, an anti-CD33 antibody drug conjugate, in the treatment of pediatric AML provides hope that anti-CD33 CAR-T cells will be effective.^28^ The degree of myeloid toxicity, the reversibility of this toxicity, and the ability to recover from this toxicity are still being studied and defined. Ongoing trials are required to further evaluate this.^28^

Studies are ongoing to create CAR-T cells that minimize the severity and duration of myelosuppression in relapsed and refractory AML.^30^ Due to the anticipated on-target/off-tumor toxicities, most trials encourage SCT following CAR-T cell therapy. Other studies are looking at using suicide genes, safety switches to be able to turn off the CAR-T cell, and genetic inactivation of specific cells.^20^^,^^31^ Currently, most CAR-T cell trials for AML aim to induce remission and bridge the patient to SCT.^24^

## Solid tumors

### Barriers

There are several barriers to solid tumor CAR-T cells that are distinct from B-cell ALL. These barriers include identifying tumor-specific antigens, heterogenous expression, inefficient trafficking, and the immunosuppressive tumor microenvironment (TME). The most ideal tumor-specific antigen to target is one that is highly expressed on tumor cells but not expressed on normal cells.^32^ This will reduce the chance of on-target/off tumor toxicity. Expression on normal cells, even if minimal, can lead to increased side effects that could be life threatening.^32^^,^^33^

Tumor-specific antigens in solid tumors can be very individualized. This individualization is often due to tumor-specific mutations that help evade the immune system.^32^ Tumor heterogeneity refers to these diverse subpopulations of cells in most solid tumors. These cell subpopulations can be different within the primary tumor, between the primary tumor and metastatic tumors, and within metastatic tumors.^34^ Due to mutations, whether somatic or germline, the same solid tumor can show diverse phenotypic and genotypic genetic differences from patient to patient. This heterogeneity can lead to varying response rates between patients and different response rates at different tumor sites within the same patient.^34^^,^^35^

**Fig. 2:**
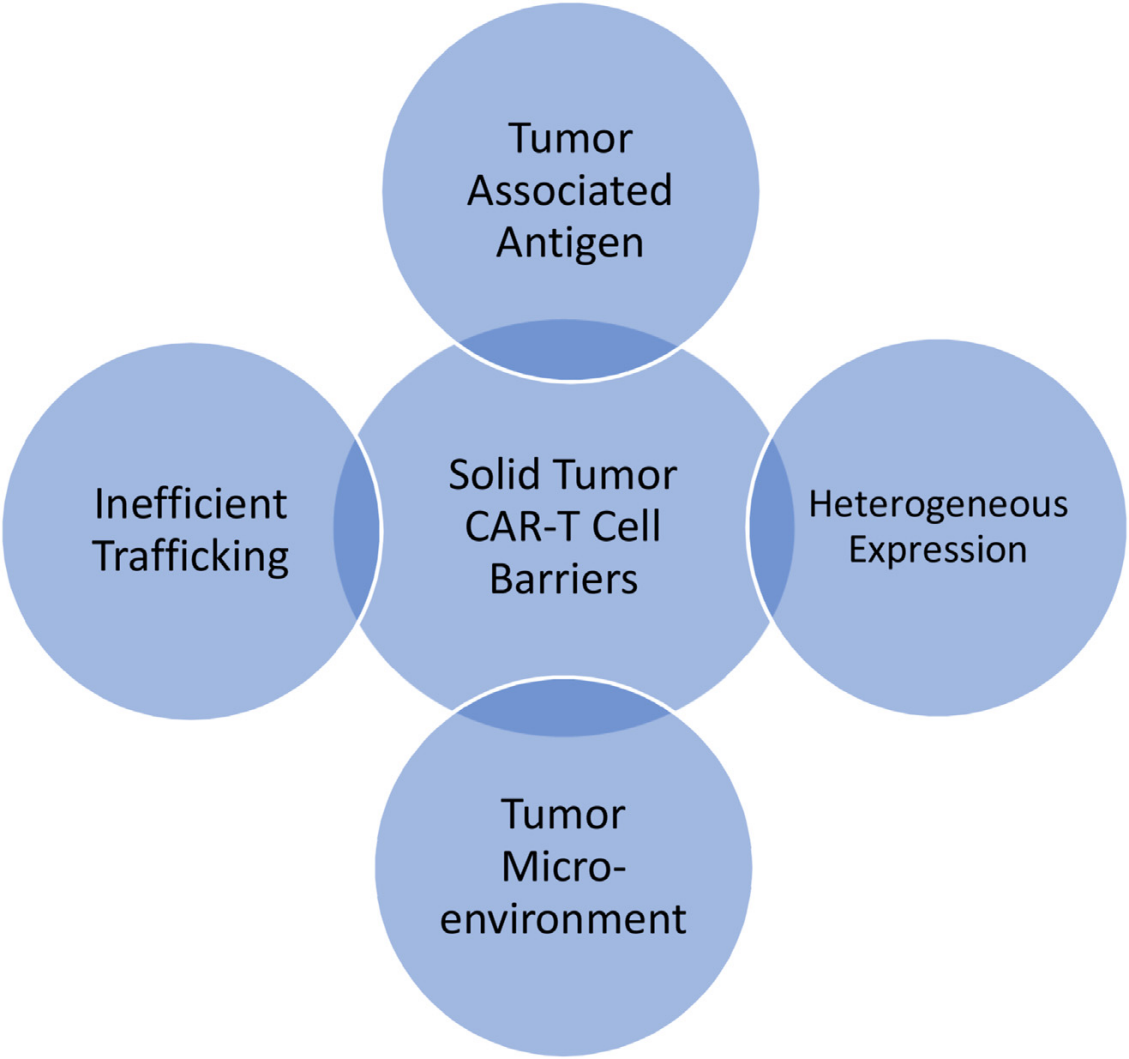
Solid tumor CAR-T cell barriers. CAR-T, chimeric antigen receptor-T.

The TME can also impede the effectiveness of CAR-T cells. The micro-environment surrounding solid tumors consists of a physical barrier of stromal cells, immune cells, and inflammatory cytokines, as well as metabolic barriers that include hypoxia and nutrient starvation.^32^, ^33^, ^34^, ^35^ The TME can impede T cell trafficking to the area as well as hinder T cells from engaging with the tumor and then proliferating.^32^, ^33^, ^34^, ^35^, ^36^ The TME also has abnormal vasculature, which can impede T cell infiltration into the tumor. The abnormal vasculature limits the delivery of nutrients and oxygen to the tumor, which leads to hypoxia, which can induce T cell dysfunction and exhaustion.^34^ A successful CAR-T cell therapy will need to address these barriers (Fig. 2).

### Neuroblastoma

Neuroblastoma is the most prevalent extracranial solid tumor in children. About 50% of patients present with metastatic disease. Though overall survival for high-risk patients has improved, it is still around 50% with chemotherapy, autologous peripheral stem cell transplant, radiation, and immunotherapy. Therefore, just as in relapsed and refractory B-cell ALL, novel therapies are needed. Neuroblastoma cells tend to be quite heterogenous; they have low expression of HLA class 1 antigens, allowing the tumor to evade the immune system, and the TME is quite hypoxic, making it highly immune suppressive.^37^

• GD2 is a disialoganglioside that is highly expressed in neuroblastoma cells. It is also expressed on neurons, peripheral nerves, and skin melanocytes.^37^ Anti-GD2 CARs have been trialed, and first-generation anti-GD2 CARs showed the product was well tolerated; however, they had poor response rates and persistence, which is most likely related to the TME.^37^ Second- and third-generation anti-GD2-CARs are being studied. These CARs have one or more co-stimulatory domains to help overcome the TME. The addition of lymphodepleting chemotherapy can also help mediate the TME. Heczey et al. did a phase 1 trial with the addition of fludarabine and cyclophosphamide prior to infusion. This trial showed increased expansion of the CAR-T cells. The chemotherapy decreased circulating T cells, allowing CAR-T cells to better expand and helping to suppress the tumor inhibitory microenvironment. While this study showed improvement in expansion, it did not show great improvement in overall effectiveness.^38^ Clinical trials are also showing nonlife-threatening side effects. A phase 1 trial of a second-generation anti-GD2 CAR-T cell treated 12 patients. Side effects of this trial included 4 patients developing grade 1–2 CRS, 3 patients with prolonged cytopenias, and 2 patients with transient neurologic side effects including headaches, blurred vision, and hallucinations. Three patients had mixed responses to tumor regression. This study shows the potential of anti-GD2 CAR-T cells with transient side effects and no on-target/off-tumor toxicities.^39^ Another phase 1 trial treated 10 patients. Side effects included grade 3–4 hematologic toxicities, grade 1–2 CRS, neuropathic pain, cough, and rash. All side effects were transient. Six patients showed stable disease at 6 months, and 4 patients had stable disease at 1 year.^40^ A phase 1–3 clinical trial using anti-GD2 CAR T-cells, a third-generation CAR that also included a suicide gene, showed favorable results with a 3-year overall survival of 60%. Twenty-seven patients were treated after receiving lymphodepleting chemotherapy. Sixty-three percent of patients showed a response at 6 weeks: 9 had a complete response and 5 had a partial response. Side effects included mainly grade 1–2 CRS, with 1 patient developing Grade 3 which was reversible with the administration of tocilizumab. One patient required the activation of the suicide gene due to altered consciousness, later determined not to be caused by CART infusion but by brain hemorrhage. This study showed that anti-GD2 CAR T-cells persisted in the bone marrow for up to two years post-infusion and in some patients for up to 12 weeks in the cerebral spinal fluid (CSF).^41^
• GPC2 is a protein that plays an important role in the growth and differentiation of axons. It is highly expressed in neuroblastoma cells but has low expression in normal cells. Murine preclinical trials have shown encouraging results for disease regression without toxicity.^42^^,^^43^
• B7–H3 is a checkpoint inhibitor highly expressed in neuroblastoma cells but restricted in normal cells. It is involved in tumor immune evasion and tumor promoting signals.^44^ Pre-clinical research is showing promise with the ability to overcome the TME.^43^^,^^45^
• LICAM (CD171) is overexpressed in neuroblastoma cells but has limited expression in normal cells. Phase 1 trials are ongoing. They have been shown to be safe with toxicities including skin rash and transient hyponatremia, but as with other CARs for neuroblastoma, they are showing limited response.^43^^,^^46^

### Brain tumors

Brain tumors are the most common non-hematologic cancers in children and are the leading cause of pediatric cancer deaths.^47^ Primary treatments include surgery, radiation, and chemotherapy. In children with developing brains, these treatments can lead to significant neurologic and neurocognitive toxicities.^47^^,^^48^ High-grade gliomas are the most aggressive brain tumors in children and include diffuse intrinsic pontine glioma (DIPG) and diffuse midline gliomas (DMG).^49^ The overall prognosis for these patients is extremely poor.^49^ Preclinical and clinical trials have shown some promise for CAR-T cell therapies.

There are specific challenges that are unique to brain tumors including the blood brain barrier, the potential for on-target/off-tumor toxicity, and tumor inflammation associated neurotoxicity (TIAN).^48^^,^^49^ The blood-brain barrier could limit the ability of CAR-T cells to cross into the CNS as it regulates the entry of immune cells into the CNS.^50^ The potential for on-target/off-tumor toxicity is related to the effects of antigen targets present on nontumor tissue. If these targets are also present in other CNS tissues, toxicities can be more harmful. For example, the GD2 antigen is located at low levels on neurons and also on peripheral nerves.^48^

Brain tumors that are located in the brainstem, spinal cord, and thalamus are sensitive to edema from inflammation.^49^ Treatment of these tumors can lead to TIAN, which is different from ICANs. This local inflammation can lead to edema, mass effects, obstruction of blood or cerebral spinal fluid flow, and increased intracranial pressure.^49^

**Fig. 3:**
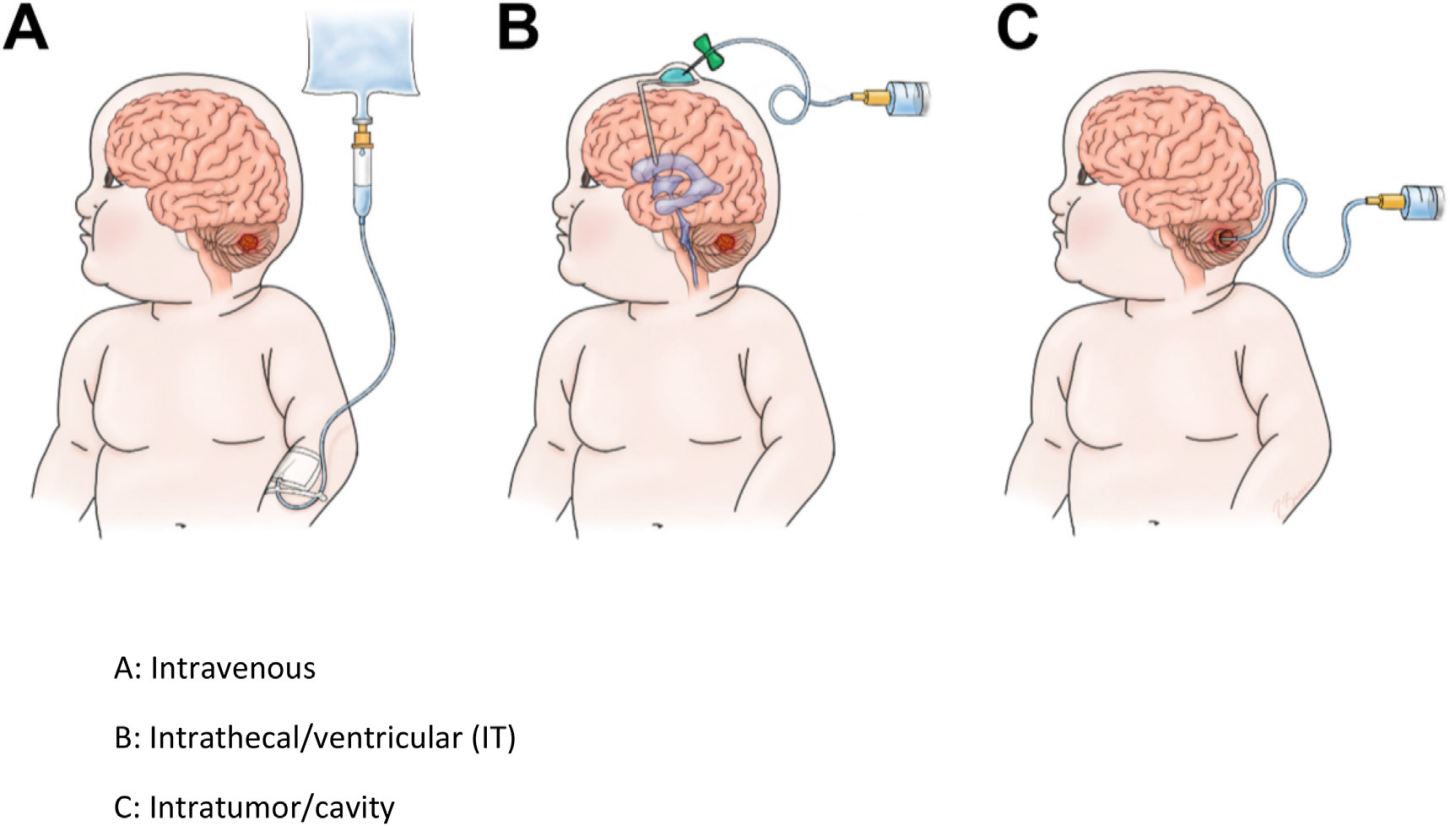
Methods of CAR-T cell delivery in pediatric brain tumors.^52^

Another difference in brain tumor CAR-T cells is the possible delivery mechanisms. Administration can be intravenous, intrathecal/ventricular, or local/regional (intratumor). Systemic administration may have limitations with CAR-T cell delivery to the tumor bed due to the blood-brain barrier. To overcome this barrier, higher doses may need to be used, which could lead to a higher risk of CRS.^51^ Intrathecal/ventricular infusions can bypass the blood-brain barrier. They can be given through a reservoir such as an Ommaya shunt, or through an existing ventriculoperitoneal (VP) shunt. Risks with this approach include infection, CSF leaks, and hemorrhage.^51^ The best method of delivery is currently unknown and still under investigation (Fig. 3).^50^, ^51^, ^52^

• GD2 is highly expressed on diffuse intrinsic pontine gliomas (DIPGs) and other diffuse midline gliomas (DMGs). It is also expressed on neurons, peripheral nerves, and skin melanocytes.^37^ Majzner et al. conducted the first phase 1 trial using IV administration in 4 patients with DIPG or spinal cord DMG. Patients developed CRS and TIAN. The TIAN was transient but led to brainstem edema, causing hydrocephalus and increased intracranial pressure. These side effects were reversible with intensive supportive care. No patient in this study experienced on-target/off-tumor side effects like neuropathies. Three of the four patients had clinical benefit.^53^
• B7–H3 (CD276) is a transmembrane protein highly expressed in brain tumors such as ATRT, DIPG, DMG, medulloblastomas, and high-grade gliomas.^54^ This protein is absent or has very low levels of expression in normal tissue.^52^ BrainChild-03 was the first human phase 1 study for repeated locoregional B7–H3 CAR-T cells in children with recurrent/refractory CNS tumors and DIPG. This study showed that repeated intraventricular infusions were well tolerated. Side effects included headache, nausea, and vomiting, all of which resolved in 72 ​h post-infusion.^54^
• Human epidermal growth factor receptor 2 **(**HER2) is found in glioblastomas. It is also expressed in epithelial tissues, skin, and muscle.^55^ An ongoing phase 1 trial of locoregional HER2 CAR-T cell therapy is showing clinical safety and feasibility of administration.^54^

## Nursing implications

CAR-T cell therapy is an evolving treatment used for hematologic malignancies and, more recently, solid tumors. The patients who receive this therapy have refractory or relapsed disease. These patients and their families experience a significant amount of stress coping with the diagnosis, in addition to traveling far for this therapy and often leaving support systems at home. Therefore, a multidisciplinary team approach is needed to provide seamless, safe care and to guide them through this process. At our institution, this team includes intake coordinators, nurse navigators, insurance specialists, inpatient and outpatient nurses, physicians, nurse practitioners, social workers, psychologists, physical and occupational therapists, nutritionists, and child life specialists. The complex care of these patients also involves successful collaboration with other departments in the hospital including interventional radiology, anesthesia, apheresis, the emergency department, the intensive care unit (ICU), neurology, and the cell and gene therapy lab. A strong research team is necessary to enable these trials to continue, and this team includes clinical research coordinators, data coordinators, research nurses, and a program manager. Clear and open communication is critical for a successfully functioning team.

Since this is an evolving therapy, it requires specialized nursing care and knowledge. Oncology nurses play a crucial role in the care of patients receiving CAR-T cell therapy from the very beginning with patient referral through the monitoring of long-term effects. At our institution, these nursing roles include nurse navigators, apheresis nurses, research nurses, inpatient bedside nurses, outpatient nurses, and nurse practitioners, all working collaboratively to meet the unique needs of this complex population of patients.

Nurses are integral to the CAR-T cell process and the success of these programs. As nurses, one of our biggest roles is that of educators. Patients and families are often very knowledgeable and have thoughtful questions regarding this therapy; therefore, nurses need to be educated and aware of the latest updates. We are responsible for the anticipatory guidance of patients and families, the education of our own staff, and those from referring institutions. Nurses need to be educated regarding who is eligible, what it is, how it works, and the short- and long-terms side effects to be able to provide education for patients and families. At our institution, we encourage our nursing staff to attend conferences at both the local and national levels. Attending conferences gives them the opportunity to expand their knowledge on CAR-T cell therapy and share what they have learned when they return. Locally, we share our knowledge through bi-annual nursing education days, where information is formally presented to nursing staff via content experts. These education days offer nurses protected time away from the unit to learn. The information is organized, and there are ample opportunities to ask questions and have educational discussions. Lectures and in-services regarding CAR-T cell therapy are also given throughout the year, especially when new trials are opening. Lectures are recorded so that new staff can watch them. Lectures are given by nurse practitioners to master's degree nursing students at the adjacent nursing school so that they are prepared when they graduate. Nurses and nurse practitioners educate and share information with colleagues at other institutions through presentations at national and international meetings, by presenting posters, and by publishing articles. As nursing pioneers in this field, it is important for us to share what we know and what we have learned.

With anticipatory guidance, our hope is to decrease anxiety and stress for patients and their families. Education is family-centered, age-appropriate, and requires an individualized approach. The establishment of a relationship with consistent nurses and nurse practitioners allows for the development of trust and comfort. At our institution, we use each interaction with patients and families as a timepoint for education. In most cases, this has been a long and complicated journey, and patients and their families have spent many years at their home institution being treated for their cancer. Families and children are often stressed by the new routines at the CAR-T cell institution, such as central line care and clinic processes. To help alleviate this stress, families are given documents that include a map for navigating the hospital, oncology-specific information like inpatient routines if admitted, phone numbers to call the clinic and to call overnight and weekends, reasons to call, written material describing CAR-T cell therapy and how it works, and suggested websites to read if they are interested.

Patients travel from all over for this therapy, and they interact with nurses throughout their entire journey. Nurse navigators are often the first contact. They are integral in collecting information to determine eligibility and maintaining communication with referrers and families. They need to discuss with the patient and family what to expect with the first visit including CAR-T cell consent conversation and T cell collection via apheresis, which often involves an apheresis catheter placement. Apheresis nurses provide education regarding T cell collection and are experts in the collection process.

Outpatient clinic nurses are the first nurses to meet patients and families in person and play a role in lymphodepleting chemotherapy education and management of side effects. Outpatient and inpatient oncology nurses are responsible for reviewing and discussing the potential side effects and monitoring for them as well. Depending on the institution, the inpatient or outpatient nurse monitors the patient on the infusion day for any infusion related complications such as anaphylaxis. These nurses are also integral in assessing for the first signs of CRS and neurotoxicity.

Bedside oncology nurses play a critical role in monitoring and assessing patients for early signs and symptoms of CAR-T cell toxicity and are at the forefront of providing supportive care.^56^ Frequent assessments are necessary to identify acute changes. Because of the potential for life-threatening toxicity, nurses must be thoroughly educated on the signs and symptoms of potential CAR-T cell related toxicities. These potential toxicities include but are not limited to tumor lysis syndrome (TLS), CRS, ICANS, and TIAN. TIAN is a newer side effect being seen with the introduction of CAR-T cell therapy for brain tumors. Nurses must know their facility's standard operating procedures for the care of patients undergoing CAR-T cell therapy. Patients receiving CAR-T cell therapies require close monitoring and vigilant nursing care during their treatment course, as prompt toxicity intervention is key to improved patient outcomes.

Cytokine release syndrome is the most common toxicity associated with CAR-T cell therapy. CRS is a systemic inflammatory response involving elevated cytokines that occurs with immune system activation. Fever is the hallmark sign of CRS, and symptoms can appear similar to those of an infection. In many patients, CRS is mild, and patients present with flu-like symptoms, including fever, chills, tachycardia, myalgias, fatigue, and headache. In contrast, other patients develop more fulminant CRS with multisystem organ failure.^57^ Severe CRS symptoms can include hypotension, dyspnea, hypoxia, respiratory distress, coagulopathies, and organ toxicities such as cardiac, renal, and liver dysfunction. A critical component of effective CRS monitoring and management is the nurse's awareness of the period during which to expect CRS development. It is recommended to anticipate onset within the first week post-CAR-T cell infusion.^58^ During this time frame, nursing care should be focused on symptom recognition such as fever or hypotension, timely intervention initiation, and symptom management. Initial treatment includes blood cultures, broad-spectrum antibiotics, antipyretics, and close vital sign monitoring. Inpatient nurses caring for this patient population must be expert and systematic in their assessments.

In addition to CRS symptoms, nurses need to be educated on how to carefully assess patients for neurologic toxicities, including headaches, confusion, tremors, ataxia, dysphasia, and seizures. Immune effector cell-associated neurotoxicity of any severity occurs in 20–60 percent of patients treated with CAR-T cell therapy.^59^ Peak ICANS symptoms occur around days 7 or 8 concurrently with or shortly after CRS following CAR-T cell infusion and resolve by days 14–21.^60^ Common early symptoms of ICANS include difficulty finding words, confusion, and impaired fine motor skills. Severe ICANS consists of seizures, coma, and cerebral edema, and in most instances, requires ICU care. Patients are proactively monitored for ICANS using institution-specific neurologic assessments by both medical and nursing teams. In brain tumor patients, TIAN results from CAR-T cell-associated inflammation at sites of disease.^53^ Nurses need to be aware of and observe for signs of increased intracranial pressure and need to be cognizant of any changes in neurological status or worsening of existing neurological deficits.

The nurse's role in the care of patients at risk for neurotoxicity after CAR-T cell therapy includes frequent assessments, symptom management, coordination of care, and timely escalation of care for patients experiencing sequelae of therapy. If signs and symptoms are recognized, neurologic assessment should occur more frequently to identify further neurologic deterioration. Supportive care includes reorientation, respiratory support, and the treatment of coagulopathies. In most cases, symptoms are transient and don't result in long-term deficits. With prompt recognition and appropriate supportive interventions, the acute symptoms of ICANS are reversible in nearly all patients.^61^

At our institution, we work very closely with our colleagues in the ICU and emergency department. We share important information about the care of our CAR-T cell patients through our unit-based nursing experts. When patients are admitted to the hospital for CAR-T cell therapy or for post-infusion complications, our unit-based clinical nurse experts reach out to the experts on the unit caring for the patient. We help to coordinate care, review side effects of chemotherapy, provide anticipatory guidance surrounding common post-infusion complications, and offer our expertise for educating and supporting patients and their families.

Early collaboration with the ICU is essential for patients developing severe CRS and ICANS. Nurse practitioners and nurses play a central role in identifying early signs of severe CRS and consulting the critical assessment team to facilitate the safe transfer of patients to the ICU if needed. Another means of collaborating with our colleagues in the ICUs is by making our high-risk patients “medical watchers”. By identifying these patients as watchers, our critical care outreach team, which consists of an ICU physician, ICU nurse, and respiratory therapist, is made aware of the patients who are at potential risk for clinical deterioration. They round on these patients twice per day and will follow their clinical status from afar. This offers both teams an opportunity for ongoing collaboration, early detection, and a smoother transfer of care should the patient become acutely and/or critically ill.

Because anti-CD19 CAR-T cells are targeted to destroy cells that express CD19, healthy B cells are also impacted. Persistence of CAR-T cells leads to B-cell aplasia, resulting in hypogammaglobulinemia, which increases the risk of infection and viral reactivation. Almost all patients who respond to anti-CD19 CAR-T cell therapy develop B cell aplasia after infusion. Immunoglobulins should be monitored and intravenous immunoglobulin replacement given until B cell function has been restored.^62^ For patients with long-term B cell aplasia (> 6 months), subcutaneous supplementation can be given at home and may be considered. Nurses must be educated about and trained in the varying methods of subcutaneous immunoglobulin administration. Studies have shown that increasing serum IgG levels are significantly associated with a lower rate of sinopulmonary infection and therefore should be monitored and proactively treated.^62^

In addition to outpatient and bedside nurses, research nurses are extremely important to the functioning of the CAR-T cell team, especially in this current era of multiple phase 1 and phase 2 trials. Some aspects of the role include ensuring the study guidelines are followed, communicating with the primary investigator, monitoring for adverse events, reporting adverse events and serious adverse events, and assuming regulatory responsibilities.

Nurse practitioners play a crucial role in the care of CAR-T cell patients and their families including providing anticipatory guidance and monitoring for and managing adverse events in the inpatient and outpatient settings. In our nurse practitioner-run clinic, nurse practitioners build rapport and relationships with the patient and family, provide support in answering questions, and help coordinate care along with diagnosing and managing adverse events. The nurse practitioner provides continuity for the patient and family and with the referring provider following CAR-T cell infusion. Education is a major responsibility, and this includes educating patients and families, educating staff at the treating institution, and being a resource for other disciplines within the institution and for outside referring providers. Inpatient and outpatient nurse practitioners provide continuity of care between the inpatient and outpatient worlds. It is imperative to share pertinent patient information such as complications and required follow-up, to ensure a seamless transition between the inpatient and outpatient settings.

**Fig. 4:**
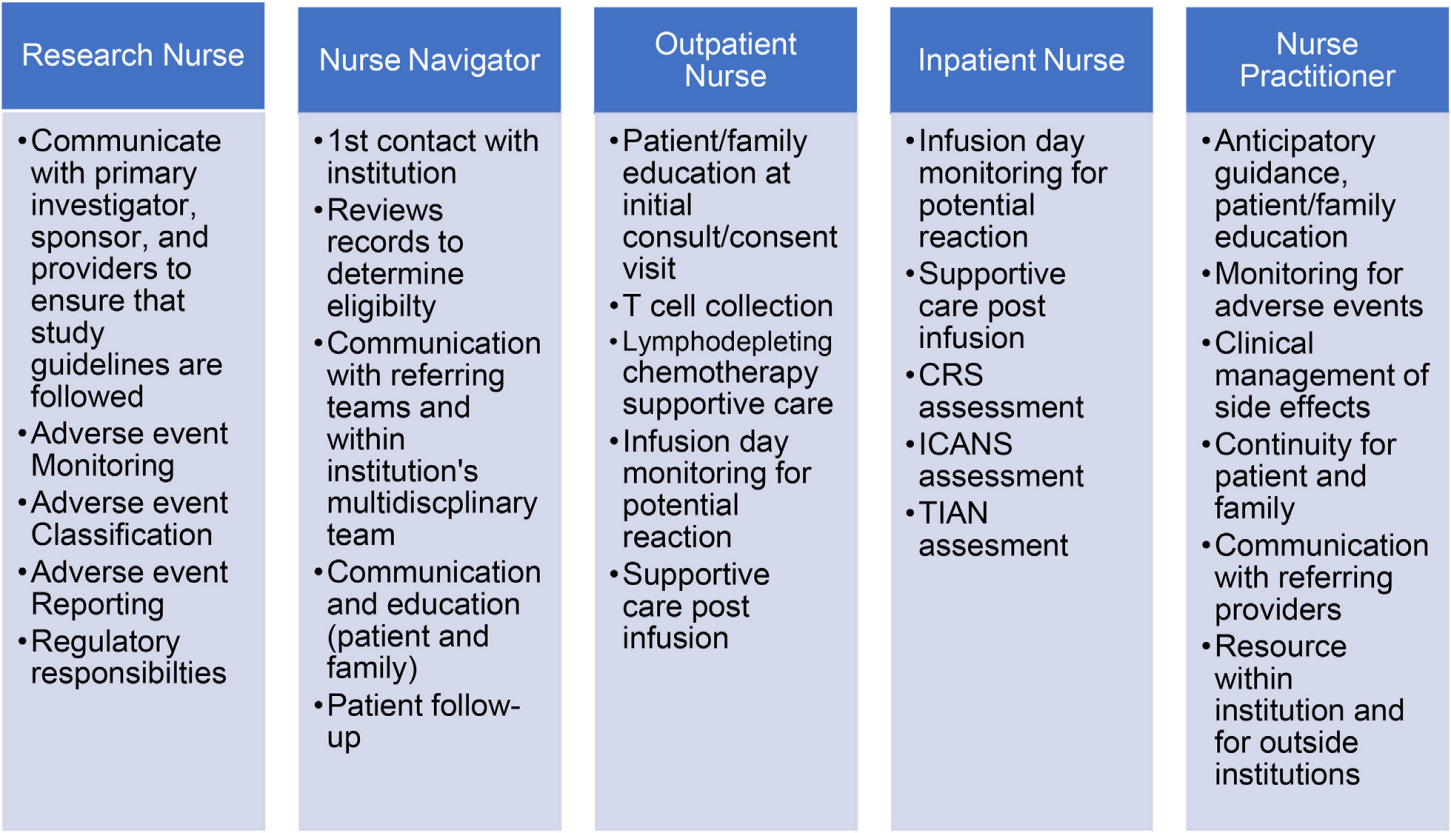
Nursing roles in CAR-T cell therapy. CAR-T, chimeric antigen receptor-T.

As CAR-T cell therapy continues to advance, this opens many opportunities for future research. Nurses are in a unique position as we are involved throughout the entire CAR-T cell process. Since nurses are so closely involved in providing anticipatory guidance and in assessing and managing side effects related to this therapy, they are at the forefront of generating research related to CAR-T cell therapy. One area of possible research is using evidenced-based practice within nursing to guide the assessment and early identification of complications such as CRS and ICANS. Patient-reported outcomes are another area that can benefit from additional research and investigation. Since this is a newer therapy, information gained in this area can be incredibly helpful in providing anticipatory guidance and symptom management to these patients during their therapy. Since this therapy has only been available for the last 11 years and the number of patients who have received it is still small, there are limited data regarding survivorship and long-term effects. This is an area where nursing research will be extremely beneficial that will need further investigation. As this therapy advances, there will continue to be numerous research opportunities for nurses to contribute to this field. Therefore, it is crucial for nurses to be educated regarding this therapy so that advances can be made, nursing practice improved, and more optimal outcomes can be achieved for patients (Fig. 4).

## Conclusions

CAR-T cell therapy has made great strides in pediatric oncology over the past 11 years. This transformative therapy began with treating patients with relapsed and refractory B-cell ALL. Although this is a promising and effective therapy for many patients, some still experience relapse. These relapsed patients require novel therapies, which led to more trials being developed for B cell ALL relapses after CAR-T cell therapy. This success has also led to trials for other relapsed and refractory pediatric hematologic malignancies and solid tumors such as neuroblastoma and brain tumors. Nurses, in various clinical and research roles, will always be at the forefront of these innovative therapies and are responsible for providing family-centered education, safe and expert patient care, anticipatory guidance, care coordination, maintaining research standards, and ongoing education of one another.

## Declaration of generative AI in scientific writing

During the preparation of this work the authors did not use AI in writing this manuscript.

## CRediT author statement

**Colleen Callahan**: original draft preparation, writing, reviewing and editing. **Lauren Haas**: original draft preparation, writing, reviewing and editing. **Laura Smith**: original draft preparation, writing, reviewing and editing. The corresponding author attests that all listed authors meet authorship criteria and that no others meeting the criteria have been omitted.
